# From genetics to migration: emerging insights into salmonid responses to environmental change from NoWPaS 2026

**DOI:** 10.1242/bio.062614

**Published:** 2026-10-02

**Authors:** Peter Betts, Hallie E. Arno, Eliot Boulaire, Samantha Crowley, Anthony Fontaine, Phoebe Kaiser-Wilks, Gabrielle Ladurée, Aglaé H. Lambert, Seán Neville, Vebjørn K. Opsanger, Joris Philip, Hayleigh Pogmore, Anna E. Silverstein

**Affiliations:** ^1^ School of Life Sciences, University of Essex, Wivenhoe Park, Colchester CO4 3SQ, UK; ^2^ Ocean Sciences Centre, Memorial University of Newfoundland, St John's, NL A1C 5S7, Canada; ^3^ DECOD (Ecosystem Dynamics and Sustainability), L'Institut Agro, INRAE, IFREMER, 65 Rue de Saint-Brieuc, 35042 Rennes, France; ^4^ Samantha Crowley, Biology Department, Dalhousie University, Halifax, NS B3H 4R2, Canada; ^5^ Université du Québec en Outaouais, Gatineau, QC J8X 3X7, Canada; ^6^ Groupe de Recherche Interuniversitaire en Limnologie (GRIL), Université de Montréal, Montréal, QC H3C 3J7, Canada; ^7^ School of Biodiversity, One Health and Veterinary Medicine, University of Glasgow, Glasgow G63 0AW, UK; ^8^ Department of Aquaculture and Fish Biology, University of Iceland at Hólar, 551 Holar, Iceland; ^9^ Centre Eau Terre Environnement (ETE), Institut National de La Recherche Scientifique (INRS), Québec City, QC G1K 9A9, Canada; ^10^ University College Cork, Cork T12 K8AF, Ireland; ^11^ Norsk institutt for naturforskning (NINA), NO-7034 Trondheim, Norway

**Keywords:** *Salmo salar*, *Salmo trutta*, *Salvelinus alpinus*, Telemetry, Physiology

## Abstract

The International Workshop of PhDs and Post-doctoral Fellows on Anadromous Salmonids (NoWPaS) provides a platform for early-career researchers to present and discuss emerging research on salmonid biology. The 2026 meeting, held in Iceland, brought together contributions spanning salmonid ecology, physiology, evolutionary genetics, conservation, and movement ecology. Here, we summarise the main themes of the meeting, highlighting how salmonids continue to serve as powerful model systems. Across sessions, studies emphasised the importance of intraspecific diversity, phenotypic plasticity, and local adaptation in shaping population dynamics, alongside advances in telemetry, genomics, and analytical approaches. Contributions also addressed key conservation challenges, including climate change, habitat fragmentation, and shifting trophic dynamics, while presenting novel tools and strategies to support management. Together, these findings reinforce the need for integrative, interdisciplinary approaches to understand and conserve salmonid populations in a rapidly changing world. The conference also facilitated holistic and collaborative research, continuing the long-held aim of NoWPaS of developing a network of early-career researchers from diverse backgrounds.

## Teaser

**Summary:** A summary of the major outcomes and progress in salmonid research presented at the 2026 NoWPaS Meeting in Iceland.

## INTRODUCTION

In a rapidly changing environment, the importance of biocomplexity (i.e. the interplay between genetics, phenotypic, and ecological diversity across and within populations) as a cornerstone to preserve natural populations has gained increasing interest over early decades of the 21st century in conservation ecology (Greene et al., 2010). In aquatic ecology, salmonids have become a model family for scientists to study the adaptive response of fish to global change. This is partly because salmonids (1) express complex and often plastic life cycles incorporating freshwater and/or estuarine, coastal and oceanic habitats (Birnie-Gauvin et al., 2019), and (2) represent a widespread economic, cultural, and natural heritage (Bolduc et al., 2025). Unfortunately, salmonids also face conservation challenges, and (3) scientists have repeatedly warned about the continuous decline of many salmonid populations over their native ranges in the past two decades (Gallagher et al., 2022).

In light of the three points outlined above, the International Workshop of PhDs and Post-doctoral Fellows on Anadromous Salmonids (NoWPaS) was established as an annual, international conference in 2005. Since then, NoWPaS annually gathers early-career (from Master’s degree to postdoctoral) researchers, in addition to keynote speakers, to present and discuss the latest scientific works regarding Salmonid research. More recently, NoWPaS has welcomed scientists from the field of evolution and genetics to widen the lens of management efforts, as salmonid conservation requires acknowledging the diversity across and, increasingly importantly, within species.

In 2026, the NoWPaS conference was held at the University of Hólar, in Iceland, a location selected for its unique location and reputation as a centre for research on phenotypic and ecological diversity in Arctic charr. A total of 31 early-career researchers and four keynote speakers (namely Professor Chris Harrod, Professor Eva Enders, Professor Arnar Pálsson and Dr Jessica Rodgers) from a range of institutions (Fig. 1), presented their current work during the following four sessions: ‘Ecology’, ‘Movement and management’, ‘Evolutionary ecology and genetics’, and ‘Conservation’ (Fig. 2). In this Meeting Review, we characterise the current state of salmonid research across the aforementioned disciplines and provide an overview of presented research themes.

## Early-career conference organising

As an annual conference organised by a committee of early-career researchers for early-career researchers, NoWPaS is a unique conference in the field of salmonid research. Each year, a new committee of volunteers is created by delegates from the conference, with a member of the previous committee acting as chair.

**Fig. 1.**
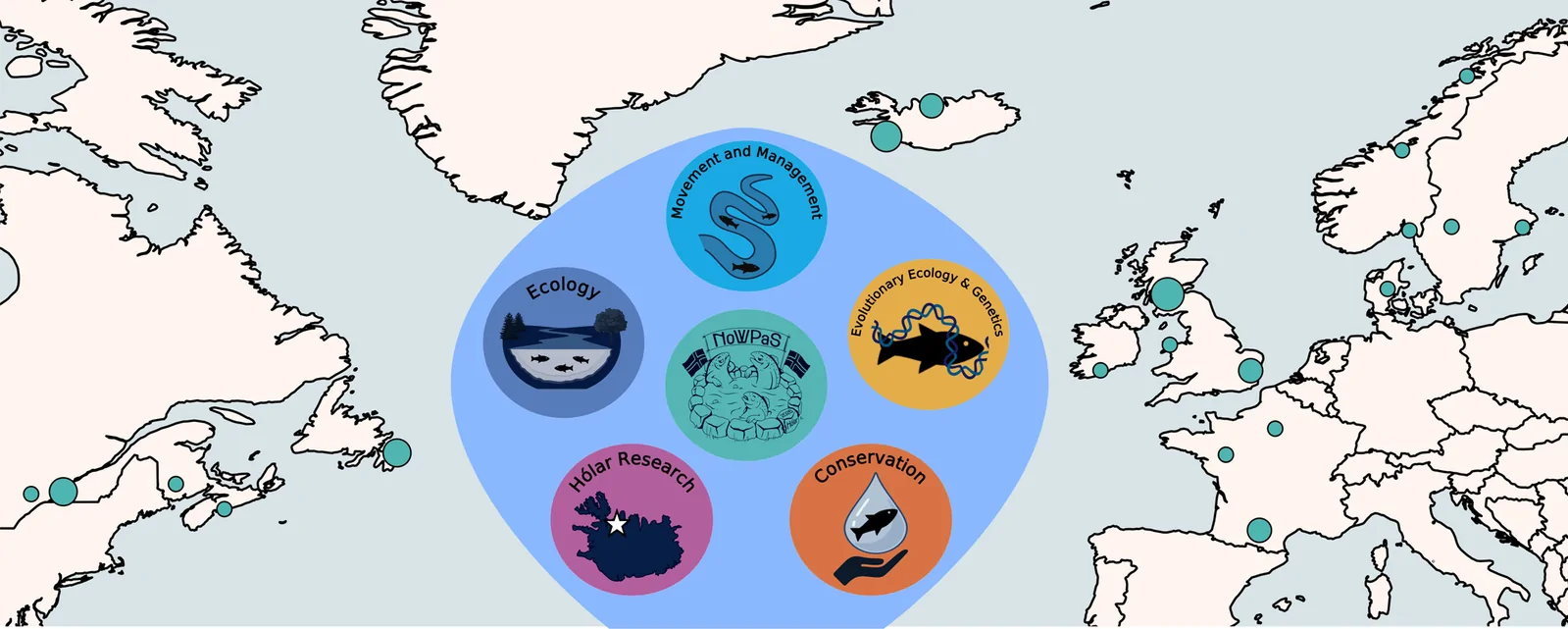
**Map showing the locations of all participants, with point sizes indicating numbers, along with the key sessions and themes for the conference.** Inset created in BioRender by Crowley, S. (2026). https://BioRender.com/27n2473. This figure was sublicensed under CC-BY 4.0 terms.

**Fig. 2.**
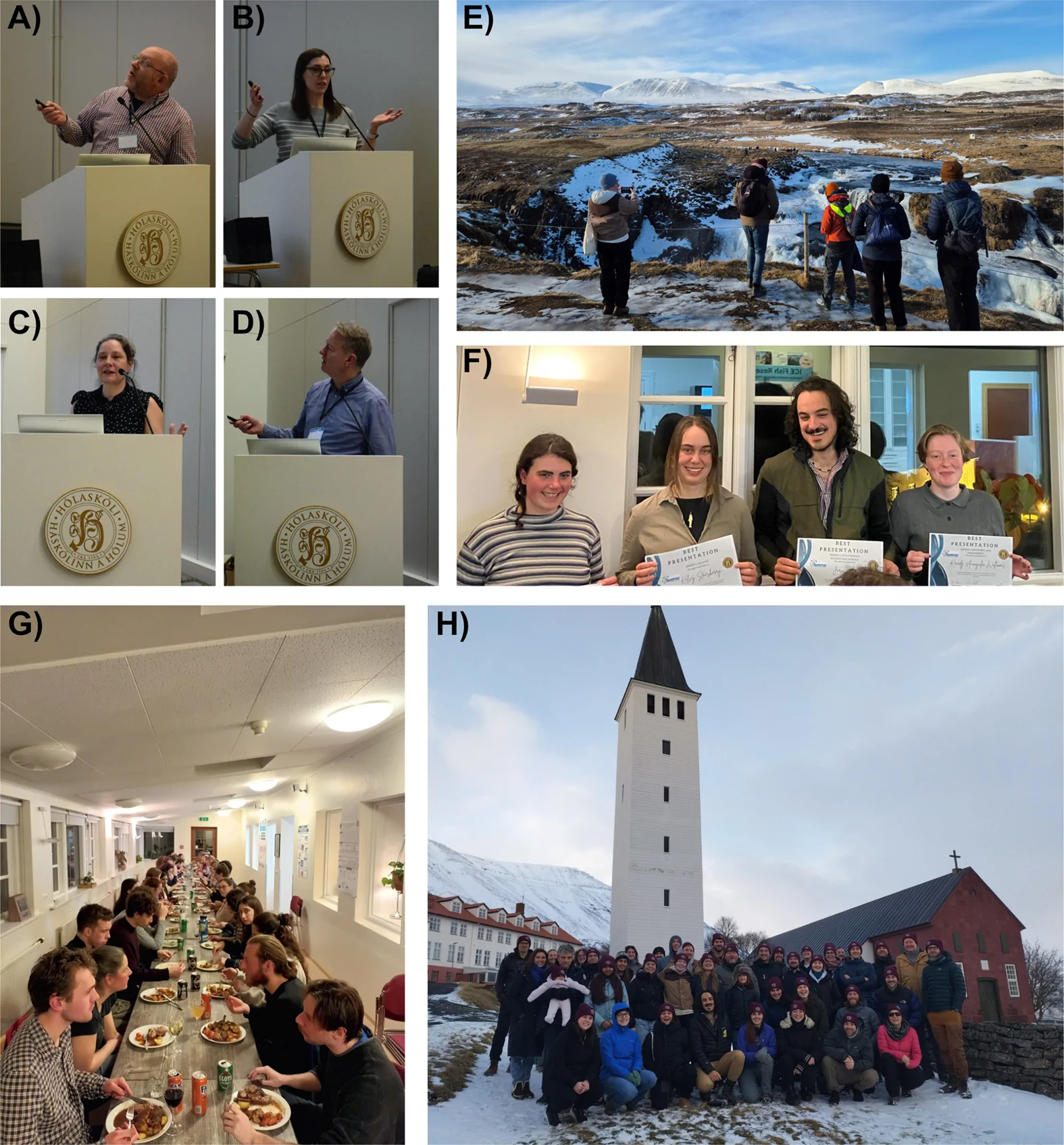
**Conference photographs.** (A-H) Photographs of the keynote speakers (A-D), prize winners (F), University of Iceland at Hólar (H), as well as all participants (E,G,H).

A core aim of NoWPaS is to give early-career researchers the opportunity to network and share their new and ongoing research on salmonids with a diverse international panel of peers. Accommodation and meals are provided for all delegates during the conference to help early-career researchers who might otherwise lack funding. It is the duty of the organising committee to secure funding, and, in doing this, committee members gain valuable experience in contacting sponsors, applying for grants, collaborating with institutions, and working within a budget. Delegates were chosen based on the relevance and significance of their abstract to the salmonid community, whilst also balancing research themes, gender, represented countries, and institutions (Fig. 3).

**Fig. 3.**
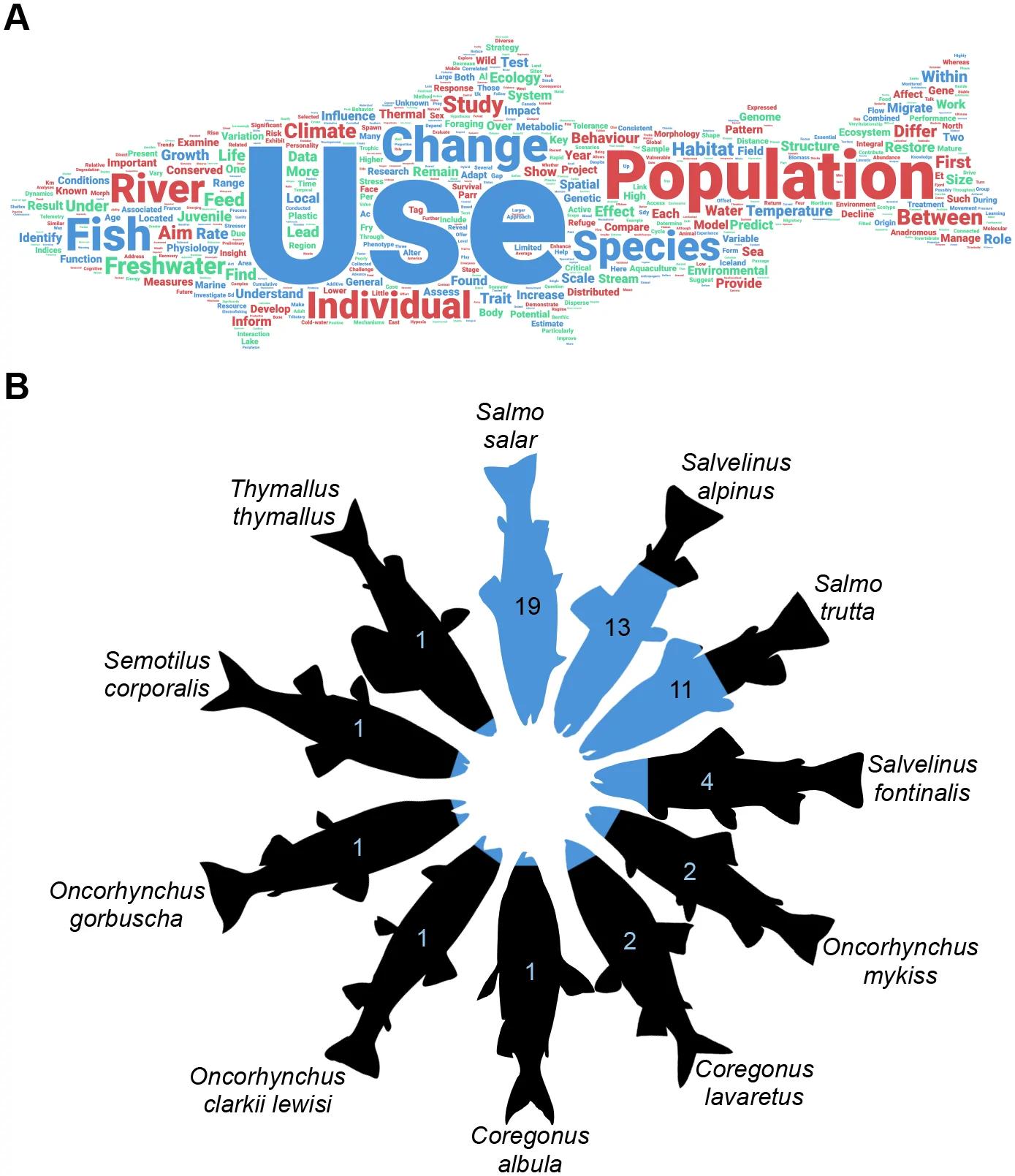
**Summary of the focus of talks at NoWPaS 2026.** (A) A word cloud generated from speaker abstracts, excluding species names. (B) Species studied by speakers. Blue shading indicates the relative proportion of each presentation devoted to a given species, normalised to the most frequently referenced species (*Salmo salar*). Numbers indicate the number of presentations in which each species was mentioned. Silhouettes derived from published sources: ^©^NOAA Fisheries, ^©^Marc Éliéser Bloch, ^©^Duane Raver and U.S. Fish and Wildlife Service, ^©^Sherman Foote Denton, ^©^Jonathan Couch and ^©^Nathaniel Moore Banta.

## Salmonid ecology and physiology

The first section of the 2026 NoWPaS conference focused on the ecology and physiology of salmonids. It opened with a keynote talk by Chris Harrod (Scottish Centre for Ecology and the Natural Environment, University of Glasgow, UK), who demonstrated how stable isotope analyses can help address different levels of ecological uncertainty. Using the framework popularised by Donald Rumsfeld's distinction between ‘known unknowns’ (questions researchers recognise but have yet to answer) and ‘unknown unknowns’ (previously unrecognised ecological processes or interactions), stable isotope data can test existing hypotheses and reveal unexpected patterns in salmonid and broader ecology. Drawing on field studies from Europe and South America, Professor Harrod illustrated how isotopic tools can bridge observational and mechanistic approaches by providing quantitative insights into diet, trophic interactions, habitat use, and energy pathways, thereby linking observed ecological patterns with the underlying processes that generate them. This integrative perspective set the stage for a session that embraced similarly integrative perspectives.

This theme was echoed in subsequent talks from delegates. Sabine Lobligeois (University of Iceland at Hólar, Iceland) presented an innovative framework to quantify personality traits in Arctic charr from the earliest life stages. By tracking individuals from hatching, her work provides rare insight into the emergence and consistency of behaviours such as activity and shelter use, and their potential implications for fitness.

Other field-based behavioural ecology studies showed that prey spatial predictability shapes dominance hierarchies and resource defence in salmonids. Analyses of space use and social interactions among sympatric juvenile salmonids revealed species-specific strategies that were temporally consistent (Kelsey Stanberry and Gabrielle Ladurée, University of Iceland at Hólar, Iceland).

Other talks examined salmonid physiological responses to environmental change. The importance of hypoxia tolerance and thermal refuges in mitigating stress in eastern Canadian salmonids was explained by Chandler Stairs (University of New Brunswick, Canada). In addition, metabolic responses to temperature were measured through respirometry and field-based estimates by Aglaé Lambert (Institut National de la Recherche Scientifique, Canada) and Peter Betts (University of Essex, UK). These were shown to shape species interactions and growth patterns across environmental gradients. In parallel, thermal tolerance was shown to vary across life stages in brown trout, pointing to uneven vulnerability within populations (Marie Pedaccini, DTU Aqua, Denmark). Novel tools to measure physiological state using machine learning and minimally invasive blood biomarkers were also demonstrated by Saad Zah (Nord Universitet, Norway).

## Evolutionary ecology and genetics

The keynote talk by Arnar Pálsson (University of Iceland, Iceland) outlined Arctic charr diversity, focusing on Icelandic populations. He highlighted the genetic differentiation among regional populations and described the use of sequencing techniques to assess these population structures, as well as contrasts between landlocked and anadromous (fish that migrate upriver from the sea to reproduce) populations. He then summarised recent work assessing the intra- and inter-ecosystem differences driven by foraging specialisation, emphasising the parallel divergence observed across the Arctic charr range into bottom foraging (benthic) and water-column foraging (limnetic) populations (Xiao et al., 2026). This theme was reinforced by Guðbörg Ósk Jóndóttir (University of Iceland, Iceland), whose geometric morphometric analyses of jaw and bone structures illustrated morphological divergence consistent with benthic-limnetic resource use. Anna Silverstein (University of Glasgow, UK) continued this by examining early-life stages and hybrids of Scottish Arctic charr to assess genetic control of divergence along the benthic-limnetic axis.

While Arctic charr exhibit extreme differentiation along this particular axis, resource polymorphism and plasticity are evident across other salmonids. Joris Philip (University of Glasgow, UK) compared differential gene expression associated with benthic and limnetic treatments in Arctic charr, brown trout, and Atlantic salmon, to determine how mechanisms of plasticity and polymorphism may be repeated across salmonids.

Behaviour and life-history traits were also addressed. Marion Dellinger (Stockholm University, Sweden) discussed behaviour syndrome through examining co-evolution between personality traits and spatial cognition in Arctic charr using common-garden experiments, linking behavioural syndromes to genetic pathways. Ruhila Goswami (University of Iceland, Iceland) highlighted the importance of the salmonid whole-genome duplication in evolutionary research, focusing on analyses of Arctic charr and brown trout to investigate variation in a sexually dimorphic Y-linked gene (*sdY*) across freshwater and anadromous populations.

Local adaptation of salmonids resulting from elevated intraspecific diversity complicates the implementation of species-level management (Fitzpatrick and Keller, 2015). Samantha Crowley (Dalhousie University, Canada) highlighted this through genomic offset modelling in Arctic charr lineages from eastern Canada. Emilio Egal [Institut national de recherche pour l'agriculture, l'alimentation et l'environnement (INRAE), France] used genome-wide single-nucleotide polymorphism analyses to associate local environmental variation with genetic structure in Atlantic salmon populations across French rivers. Hallie Arno (Memorial University of Newfoundland, Canada) then explored the consequences of genetic mixing, known as introgression, between wild and aquaculture salmon populations, highlighting how introgression may affect local climate adaptation of wild populations due to maladapted traits not under selection pressure in aquaculture environments and, therefore, affect their resilience to rapidly changing environments.

## Salmonid conservation

Understanding the drivers of salmonid decline was a key focus within the conservation session. Professor Eva Enders (Institut National de la Recherche Scientifique, Canada) highlighted her research on informing recovery strategies and identifying suitable habitat for salmonids under a changing climate. Collectively, her work demonstrates how conservation physiology approaches, and integrating individual heterogeneity via respirometry, telemetry, and behavioural assays, can better inform evidence-based management of these vulnerable species.

Patterns in, and factors driving, salmonid mortality were explored in detail. Phoebe Kaiser-Wilks (University of Glasgow, UK) analysed long-term catch data from the International Council for the Exploration of the Sea (ICES), an organisation responsible for international fish stock assessments. This analysis highlighted the overall decline of Atlantic salmon populations between 1973 and 2021. Declines varied across spatial scales and life-history strategies; this set the stage to explore more of the detail driving mortality. Eliot Boulaire (UMR DECOD, Institut Agro Rennes-Angers, France) discussed a positive correlation between marine survival of Atlantic salmon smolts and body size, but at the cost of early maturing, leading to lower individual fecundity. His research highlights the importance of understanding individual heterogeneity when managing populations. Mikael Ranta (Memorial University of Newfoundland, Canada) discussed how variation in prey availability can strongly influence the dynamics of juvenile Atlantic salmon, emphasising the role of trophic constraints in shaping population trajectories.

A range of innovative conservation strategies are being developed to address threats faced by salmonid populations. Hugo Gallier (Institut National de la Recherche Scientifique, Canada) introduced the Cumulative Effect Model for Prioritizing Recovery Actions (CEMPRA), which integrates land-use and climate scenarios to better predict salmon population dynamics. In response to the critical threat of habitat fragmentation, Niclas Carlsson (Karlstad University, Sweden) presented ongoing evaluations of Fishheart, a fish-passage system designed to help aquatic life safely bypass artificial barriers. The system enables fish to reach key habitats and restores connectivity between rivers and the sea in waterways heavily shaped by man-made infrastructure.

Patrizio Feliziani (Bangor University, UK) spoke about his work developing a multi-pathogen environmental DNA (eDNA) toolkit to improve the detection of priority pathogens, thereby supporting more effective management of disease risks. Nature-based solutions were also emphasised; Hayleigh Pogmore (University of Essex, UK) spoke about their research investigating how riverbank vegetation buffers stream temperatures and enhances the resilience of brown trout dominated Spanish streams to climate warming.

## Migration, management and habitat use

The key theme that emerged during this session was the importance of population-level data to identify threats and inform effective management practice. Jessica Rodger (University of Glasgow, Atlantic Salmon Trust, UK) was the keynote speaker for the session. Her work focused on the use of acoustic telemetry to address critical knowledge gaps in the marine migration phase of post-smolt Atlantic salmon in coastal and offshore waters of the UK and Ireland. Over 1000 smolts from rivers on the west coast of Scotland, Northern Ireland, England and Ireland were tagged and tracked as they moved through coastal and offshore waters. Acoustic telemetry data on the migration pathways, timing and survival of Atlantic salmon during this highly vulnerable life stage can be used to inform policy, environmental impact assessments and fishing regulations. These approaches were also used by Håvard Frafjord (Norwegian University of Science and Technology, Norway) to investigate the effects of coastal development on sea trout movement and foraging. Ava J. Hart (Memorial University of Newfoundland, Canada) investigated the effects of biological traits such as fork length – a commonly used length measurement for salmonids (defined as the distance from the tip of the snout to the deepest point of the forked tail fin), lipid content, sex and reproductive history on upstream migration distances of Atlantic salmon. These findings all highlighted the considerable variation in distance and pathways followed by migrating salmonids.

The effects of anthropogenic climate change on salmonid habitats were also explored. Kenedy Annejulee Williams (University of Iceland, Iceland) discussed the decline of Arctic charr catch in Iceland and the potential vulnerability of river spawning anadromous Arctic charr to climate change. Maxime Descat (INRAE, France) investigated how extreme hydrological events such as floods and droughts influence the functional morphology of salmonids. Quentin Josset (French Biodiversity Agency, France) then put these modern stressors into historical context by examining determinants of age at first return in sea trout.

Complementing these themes, there was a clear applied management focus in several talks. Anthony Fontaine (Université du Québec en Outaouais, Canada) examined whether rivers that produce a high proportion of juvenile Atlantic salmon also contribute proportionally to the number of returning adults. Seán Neville (University College Cork, Ireland) presented a novel population assessment method for juvenile salmonids in shallow streams using underwater video, a non-harmful year-round survey tool. Finally, Vebjørn Kveberg Opsanger (Norwegian Institute for Nature Research, Norway) reviewed the effectiveness of river restoration for improving juvenile salmonid habitat. He summarised studies in Norway from 1993-2024 showing that restoration projects had a significantly positive effect.

## Iceland as a crossroads for research on salmonids

On the final morning of the meeting, Bjarni Kristófer Kristjánsson, Skúli Skúlason, David Ben Haïm, Camille Leblanc and Stefán Óli Steingrímsson, representing the Department of Aquaculture and Fish Biology at University of Iceland at Hólar, presented their research. A central theme to this session was that biodiversity within species, expressed through variation in morphology, behaviour, and life histories, can equal or exceed differences among species. This point is particularly striking within low-diversity and species-rich Icelandic freshwater systems, where large genetic and phenotypic variation is seen within and among ecosystems. Together, these patterns reinforce the importance of conserving whole ecosystems and their dynamic processes, rather than focusing solely on conserving individual taxa.

Empirical work presented across systems demonstrated how ecological drivers such as food availability, population density, and hydrodynamic conditions influence behaviour and resource use in stream-dwelling salmonids. At the same time, behavioural traits, and consistent individual differences often described as personalities, were shown to have a strong genetic basis and to persist across contrasting environmental contexts.

Variation among salmonid populations was further framed within an eco-evo-devo approach, where environmental heterogeneity influences developmental trajectories that feed back into evolutionary divergence, particularly in systems characterised by repeated ecological opportunity and strong selection pressures (Skúlason et al., 2019). Long-term and seasonal dynamics emerged as critical considerations for interpreting patterns of growth, trophic interactions, and population structure, underscoring the need for temporally explicit ecological sampling and analysis.

The work of scientists at University of Iceland at Hólar illustrates how the studies of Icelandic salmonids contribute to our understanding of evolution of species as a whole, from studying individual traits to responses of populations to environmental fluctuations. These contributions illustrated how Iceland is an ideal place for researchers to test hypotheses, and how Icelandic research expertise effectively bridges ecological theory, long-term empirical studies, and conservation, offering a coherent framework for understanding and managing salmonids' diversity in a rapidly changing world.

## Conclusion

The 2026 NoWPaS conference emphasised that understanding salmonid responses to environmental change requires integrating scales of biological organisation from genes to ecosystems. Across sessions, contributions showed that population dynamics emerge from interactions among individual variation, local adaptation, and environmental context, supported by advances in telemetry, genomics, and statistical tools.

Collectively, these findings indicate that effective conservation must move beyond protecting habitat and species-level biodiversity to also preserve intraspecific genetic and phenotypic diversity, such as the distinct morphs that perform differentiated ecosystem functions within a single species. Interdisciplinary approaches and continued early-career collaboration will be essential to improve management under accelerating environmental change. NoWPas will continue with another conference scheduled for 2027, continuing this valuable network for early-career researchers.
